# Increasing access to palliative care for patients with advanced cancer of African and Latin American descent: a patient-oriented community-based study protocol

**DOI:** 10.1186/s12904-023-01323-0

**Published:** 2023-12-20

**Authors:** Anna Santos Salas, Sharon M. Watanabe, Aynharan Sinnarajah, Nahyeni Bassah, Fleur Huang, Jill Turner, Jacqueline Alcalde Castro, Hannah M. O’Rourke, Pilar Camargo-Plazas, Bukola Salami, María Santana, Katy Campbell, Omar Abdel-Rahman, Tracy Wildeman, Lisa Vaughn, Harkeert Judge, Sadia Ahmed, Bisi Adewale, Iqmat Iyiola, the Patient Advisory Council, Nazret Russon, Atobrhan Godlu, María Castrellon Pardo, German Mendez Mendez, Edna Ramirez, Tibebe Weldehanna, Foto Asfaha, Meron Seyoum, Brenda Cameron, Bayo Oladele, Yinka Oladele

**Affiliations:** 1 Faculty of Nursing, College of Health Sciences, University of Alberta, Third Floor Edmonton, Clinic Health Academy, 11405 87 Avenue, Edmonton, AB T6G 1C9 Canada; 2 Division of Palliative Care Medicine, Department of Oncology, Faculty of Medicine and Dentistry, College of Health Sciences, University of Alberta, 11560 University Avenue Edmonton, Alberta, T6G 1Z2 Canada; 3 Department of Medicine, Queen’s University, 34 Barrie Street, Kingston, ON K7L 3N6 Canada; 4 Division of Radiation Oncology, Department of Oncology, Faculty of Medicine and Dentistry, College of Health Sciences, University of Alberta, 11560 University Avenue, Edmonton, AB T6G 1Z2 Canada; 5 Supportive Care Team Cross Cancer Institute and Division of Palliative Care Medicine, Department of Oncology, Faculty of Medicine and Dentistry, College of Health Sciences, University of Alberta, 11560 University Avenue, Edmonton, AB T6G 1Z2 Canada; 6 Department of Psychosocial Oncology and Supportive Care, Princess Margaret Cancer Centre , Department of Medicine, University of Toronto, 610 University Avenue, Toronto, ON M5G 2M9 Canada; 7 School of Nursing, Queen’s University, Kingston, ON K7L 3N6 Canada; 8 Cumming School of Medicine, University of Calgary Foothills Campus, 3330 Hospital Drive NW, Calgary, AB T2N 4N1 Canada; 9 Patient Engagement Team, Alberta SPOR SUPPORT Unit, 3280 Hospital Dr NW, Calgary, AB T2N 4Z6 Canada; 10 Department of Women and Gender Studies, Faculty of Arts, College of Social Sciences and Humanities, 3-51 Assiniboia Hall, Edmonton, AB T6G 2E7 Canada; 11 Medical Oncology Cross Cancer Institute and Department of Oncology, Faculty of Medicine and Dentistry, College of Health Sciences, University of Alberta, 11560 University Avenue, Edmonton, AB T6G 1Z2 Canada

**Keywords:** Advanced cancer, Palliative care, Black, Latino, Health services accessibility, Healthcare disparities, Patient engagement

## Abstract

**Background:**

Cancer disparities are a major public health concern in Canada, affecting racialized communities of Latin American and African descent, among others. This is evident in lower screening rates, lower access to curative, and palliative-intent treatments, higher rates of late cancer diagnoses and lower survival rates than the general Canadian population. We will develop an Access to Palliative Care Strategy informed by health equity and patient-oriented research principles to accelerate care improvements for patients with advanced cancer of African and Latin American descent.

**Methods:**

This is a community-based participatory research study that will take place in two Canadian provinces. Patients and community members representatives have been engaged as partners in the planning and design of the study. We have formed a patient advisory council (PAC) with patient partners to guide the development of the Access to Palliative Care Strategy for people of African and Latin American descent. We will engage100 participants consisting of advanced cancer patients, families, and community members of African and Latin American descent, and health care providers. We will conduct in-depth interviews to delineate participants’ experiences of access to palliative care. We will explore the intersections of race, gender, socioeconomic status, language barriers, and other social categorizations to elucidate their role in diverse access experiences. These findings will inform the development of an action plan to increase access to palliative care that is tailored to our study population. We will then organize conversation series to examine together with community partners and healthcare providers the appropriateness, effectiveness, risks, requirements, and convenience of the strategy. At the end of the study, we will hold knowledge exchange gatherings to share findings with the community.

**Discussion:**

This study will improve our understanding of how patients with advanced cancer from racialized communities in Canada access palliative care. Elements to address gaps in access to palliative care and reduce inequities in these communities will be identified. Based on the study findings a strategy to increase access to palliative care for this population will be developed. This study will inform ways to improve access to palliative care for racialized communities in other parts of Canada and globally.

**Supplementary Information:**

The online version contains supplementary material available at 10.1186/s12904-023-01323-0.

## Background

Socioeconomic inequalities are a rising public health concern in Canada [1, 2]. Racialized groups in Canada bear disproportionately high rates of socioeconomic inequalities and are prone to health disparities [1]. Gaps in access to cancer diagnosis and treatment affecting persons living in low income areas, immigrants, and rural and remote populations exist in Canada [3]. These result in higher rates of advanced cancer diagnoses among members of these population groups [1, 4–7]. Marked cancer disparities affecting members of these populations exist in other countries [8–10]. Evidence of cancer disparities affecting racialized communities of African and Latin American descent is limited in Canada [11]. Available studies have focused on access to screening and use population-wide or immigrant data, with limited Black and Latin American representation [11]. There is a need to improve equity in access to palliative care and understand the palliative care experiences of racialized populations. These populations face severe health inequities because of barriers in accessing care, precarious income, employment, housing, transportation, negative health care encounters, and experiences of racism and discrimination [1, 12, 13].

Blacks and Latin Americans represent 15.6% and 5.8% of the racialized population in Canada, respectively [14]. They bring a rich history of cultural traditions, diversity, knowledge and skills to Canadian society. The majority of Blacks report Africa as their region of birth [15]. Edmonton and Toronto are among the top 5 urban areas with high numbers of people of African descent [15] and The prairies have the fastest growing population of Blacks in Canada [16]. In Alberta, the Black population quintupled their size between 1996 and 2016 [15]. In the early 2000s, Latin Americans represented close to 1% of the population [17]. In 2016, they were about 2% of the Canadian population, with 674,640 individuals [18]. The majority of people of Latin American descent had been born outside of Canada in 2001. In 2001, Ontario, Quebec, British Columbia, and Alberta had the majority of people of Latin American descent [17]. In 2016, racialized groups had a higher unemployment rate than non-racialized Canadians (9.2% vs 7.9%) [14]. Serious socioeconomic inequalities are reported for members of African and Latin American communities [1, 19].

### Socioeconomic inequalities in cancer care

Reports in the United States reveal cancer disparities affecting African Americans and Hispanics [10]. Compared to their white counterparts, cancer incidence is 6% higher in Black men while cancer mortality is 19% higher. In Black women, cancer incidence is 8% lower and cancer mortality is 12% higher than in white women. Black Americans have the highest rates of advanced cancer diagnoses for common cancers [8]. Latinos have a high incidence and mortality of liver, stomach, and cervical cancers [10], are less likely to be diagnosed at an earlier cancer stage, and have a lower 5-year relative cancer survival rate than Whites [9]. Socioeconomic inequities in palliative care have been found in Canada [20–22]. An Ontario study of end-of-life cancer care found that immigrants from racialized groups had higher rates of aggressive end-of-life care than white immigrants. Blacks were among those with the lowest likelihood of receiving supportive care [23].

Palliative care promotes quality of life and wellbeing for individuals with advanced illness [24, 25]. Early palliative care improves clinical outcomes [26] and symptom relief [27], reduces aggressive care at the end-of-life, and may improve survival [28]. Guidelines recommend the provision of early palliative care to begin within a few weeks of diagnosis of advanced disease [27, 29]. Studies of early palliative care in underserved populations are few [30, 31]. Patient navigation is an innovative model to improve patients’ experiences [32, 33], and reduce cancer disparities [34–39]. Patient navigators provide personalized care and improve access to health care [34, 40, 41]. A patient navigation intervention improved access to early supportive and palliative care for advanced cancer patients in Mexico [42]. A meta-analysis of patient navigation interventions suggested patient navigation increases the likelihood of access to care [43]. Together with our Patient Advisory Council (PAC), we will explore how patient navigation can assist to address inequities in access to palliative care.

Reviews focusing on Blacks in the United States and Canada report barriers in access to cancer care including low income, racism and discrimination, multiple jobs, family caregiving, lack of trust in the health care system, lack of Black health care providers, and difficulties navigating health care [44, 45]. Similar issues have been found in studies including African American or Hispanic women [46–48]. A study of Latino patients with metastatic advanced cancer in the United States revealed many patients believed their cancer was curable or that they had early-stage cancer [49]. Patients who thought their cancer was curable expressed a preference for life-prolonging treatments. Among patients who knew their cancer was incurable, they expressed a desire to access palliative home care, being pain free, prioritize comfort care, and involve their families in their end-of-life decisions [49]. These studies show the need to increase awareness of as well as access to palliative care services in ways that are tailored to the sociocultural characteristics of this patient population. Research on the experiences of patients with advanced cancer of African and Latin American descent can shed light into what needs to be done to improve their access to palliative care in Canada. There is a lack of qualitative studies on the cancer care experiences of people of African and Latin American descent in Canada.

### Study aims

The purpose of this study is to develop an access to palliative care strategy informed by early palliative care and patient navigation to accelerate access to palliative care for patients with advanced cancer of African and Latin American descent. Our specific aims are:

1. To explore the access to palliative care experiences of advanced cancer patients of Latin American and African descent;
2. To engage advanced cancer patients of African and Latin American descent, their families, and communities to delineate together an access to palliative care strategy; and
3. To develop an implementation plan together with patients, families, and health care providers.

### Design and methods

We will undertake a community-based participatory research (CBPR) study. An intersectionality lens will also inform the study. Intersectionality points to the role of power structures in producing experiences of dominance and subordination [50]. People who are subject to these power dynamics are marginalized in all aspects of society, including health care [51]. Rooted in Black feminist thought, intersectional theory conveys how social dimensions related to race, class, gender, sexual orientation, disability (and other identity markers) interact, amplify, and shape people’s lived experiences [52–56]. The emancipatory potential of CBPR can be empowering [57]. Rooted in social justice, CBPR typically combines the agenda of research, education, and social action to facilitate conditions for both individual and community change [58]. It provides a promising approach to address social inequities by emphasizing reciprocal and equitable relationships with community partners [59].

We are enacting our community-based approach by actively engaging members of the Latin American and African communities through a patient engagement process. Patient partner is an inclusive term that refers to people with lived experience of a particular health situation, their families, or friends [60]. Involvement of patient partners in cancer and palliative care has shown positive results such as living well with cancer, personal growth, and accessing new opportunities [61]. Following the Canadian Institutes of Health Research patient engagement principles namely, inclusiveness, support, mutual respect, and co-build [60], we have begun a process of reciprocal collaboration with patient partners. Patient partners are patients with advanced cancer, family members, or community members who self-identify as being of African or Latin American descent. We have started a PAC with eight expert patient partners and have discussed and refined our study plan. They bring a rich history of involvement in other initiatives and several have research experience in patient and community engagement or have served as patient advisors. We are collaborating with the African Cancer Support Group (ACSG) in Alberta and are approaching other African and Latin American community organizations to enhance the community engagement component of the study. According to the Strategy for Patient Oriented Research, a patient partner is a patient who is involved in a research project in a capacity other than as research participant [60]. They are members of the study team and have an active role in the design and implementation of the study. Given the health status of our study population, we may not be able to engage patients with advanced cancer as patient partners. None of the patient partners will be research participants and research participants will not be invited to become patient partners in this study. The PAC will oversee the study and provide guidance on study processes.

### Settings and sample

We will have three study sites including one site in Edmonton, Alberta and two sites in the Greater Toronto area in Ontario. In Edmonton, we have secured site approvals with three clinical teams involved in the care of advanced cancer patients at the Cross-Cancer Institute (CCI), a large tertiary cancer centre serving the northern half of the province. These teams include Symptom Control and Palliative Care, Palliative Radiation Oncology, and Supportive Care. The CCI serves diverse populations in the province. Participating sites in Ontario include the Palliative Care department at a large tertiary cancer centre and one of the health regions in the province serving diverse populations. Research participants will be patients with advanced cancer, their caregivers, or community members who self-identify as being of African or Latin American descent and who have had lived experience of cancer; or have been close to someone with cancer; or have an interest in increasing access to palliative care. Spouses or caregivers who are not of African or Latin American descent will also be eligible to participate. We will engage providers of African or Latin American descent, or health care providers who have worked with these populations and are involved in the care of patients with advanced cancer.

We will employ purposive sampling and will aim for a sample size of 100 participants. We plan to engage 20 patient-family dyads in each province (40 dyads in total), or 80 patients and family members, 10 community members, and 10 health care providers. We will aim for similar numbers of participants of African or Latin American descent. Since we are interested in learning from the experiences of two racialized communities, we anticipate this sample size will be sufficient to achieve an in-depth understanding of their lived experiences [62]. An honorarium of $35 will be provided to each patient participant and a $25 gift card to health care providers as a gesture of appreciation for their contributions. From an ethical perspective, this amount is unlikely to result in coercion.

### Participant recruitment strategy

One method of patient recruitment will be to approach patients and families through the clinical teams at participating sites. We have co-designed a poster with our patient partners that will be placed in the waiting room of our study sites. A member of the clinical team will serve as an intermediary for the initial contact of potentially eligible participants. The intermediary will briefly describe the study following a script and eligible participants will receive a letter of initial contact. Informed consent will be sought for the release of personal information to a study team member who will contact them to explain the study and obtain written or virtual informed consent. A second method involves, patients and families contacting the study team directly via phone or email (contact information will be on the poster). With participants who may be unable to provide written informed consent, we will request the involvement of a member of the clinical team to serve as an impartial witness. We will also use word of mouth through study participants or patient partners for the accrual of additional patients, family members, or community members. Posters will be in English and include a note that we may be able to interview in Spanish and selected African languages. For accrual of providers, we will send emails with study information to the clinical teams via their team administrators. Providers will contact a member of the study team via email who will then begin the informed consent process.

### Data collection

Data collection is described by aim:

#### Study aim 1: Explore the access to palliative care experiences of participants

We will conduct in-depth interviews with patient-family dyads and community members to delineate the experiences of access to palliative care of advanced cancer patients. We will interview patients and families together to develop a relational understanding of their lived experience, unless they prefer otherwise. In every case, we will consider the patient-family dyad as a unit and examine their data together. The interviews will last 60 min and will take place via phone, videoconferencing, or in person depending on participants’ preferences. They will occur at a time and place convenient to participants. We have developed and refined an interview guide together with our patient partners. We will aim for a few questions to facilitate dialogue and a focus on participants’ experiences. Please see interview guide for details (Supplementary file 1). We will also explore participants’ perspectives concerning the intersections of race, gender, socioeconomic status, immigration status, language barriers, housing status, place of residence, transportation, and other social categorizations to elucidate their role in their access experiences. Interviews will be digitally recorded and transcribed verbatim. In addition, we will collect voluntary self-disclosed socio-demographic information through anonymous online surveys with patients and family members. We will ask patients basic information regarding their cancer diagnosis and treatments (see patient interview guide). We will also conduct an online anonymous survey to collect professional background and work-related information from health care providers.

Although people may be comfortable speaking English, we will offer the option of interviewing participants in their home language when feasible. Data show that about a quarter of the Black population in Canada [15] and about 10% of the Latin American population [17] are not fluent in English or French. We will not recruit participants unable to understand the English consent form unless we are able to secure the assistance of a qualified oral translator/interpreter. We will offer the option of interviewing in Spanish to Spanish-speaking participants of Latin American descent and may be able to interview members of African descent in selected African languages. We have several study team members fluent in African languages or Spanish. Interviews conducted in Spanish or other languages will be transcribed into the original language and then translated into English by the research assistants themselves or by the transcription company that we engage.

#### Study aims 2 & 3: Delineate together an Access to Palliative Care Strategy and develop an implementation plan

In parallel to study activities for aim 1, we will begin the development of a patient-oriented Access to Palliative Care Strategy for racialized populations together with our patient partners from the PAC. The degree of health care provider engagement in the delineation of the strategy will be decided with the PAC. The strategy will be informed by research findings from study aim 1, team expertise, evidence from the literature, and the guidance of our patient partners. We will seek to incorporate diverse types of knowledge. First, we will integrate knowledge of participants’ experiences of living with advanced cancer, their experiences of access to palliative care, and health inequities. Second, we will incorporate knowledge of early palliative care by exploring the impact of early access to palliative care, and potential challenges and mitigation strategies to facilitate access. Third, we will examine patient navigation as a validated approach to remove barriers in access to care with underserved communities. These will be achieved through interviews with study participants as described above, and PAC meetings. Our patient partners have voiced the difficulties of navigating the cancer care system experienced by members of their communities due to language barriers, lack of knowledge of the Canadian health care system, and negative experiences with the health care system. Although it is early to determine how patient navigation will be integrated into the strategy, we believe learnings from patient navigation can inform the crafting of key elements of the strategy. Patient navigation has been shown to be a successful approach to increase access to cancer care [63–66]. In the early stages of this study, our patient partners voiced the need for patient navigators in the cancer care system for racialized populations. The PAC will meet on a monthly and ad hoc basis until the end of the study. We will follow an iterative dialogical process to delineate the strategy. Freire called this process *cultural circles* where participants define and develop key themes together [67–69].

Once we achieve a draft of the strategy, we will organize a series of four 2-h virtual conversational meetings to examine the appropriateness, effectiveness, risks, requirements, and convenience of the strategy together with patient partners, study implementers, and providers. We have developed the consent form and a preliminary question guide for health care providers to undertake this research activity (Supplementary file 2). The virtual conversations will provide an opportunity to request feedback and make revisions to the strategy as needed. Our study implementers will provide input concerning feasibility, requirements, and challenges from a health system perspective and facilitate liaisons with stakeholders. Our patient partners will bring the patient perspective as delineated in study findings that will be informed by their own lived experiences. We will also invite a sample of 10 health providers to share their perspectives concerning the strategy and steps for a successful implementation. Since the virtual conversations will take place in the second year of the study after we develop the draft Access to Palliative Care Strategy, we may need to request a study modification with a revised question guide and consent form for health care providers. These virtual conversations will be digitally recorded and transcribed verbatim. We will summarize the data and make revisions to the strategy based on these discussions. The strategy will include key implementation facilitators and barriers to overcome based on our discussions with patient partners, implementers, and providers. The development and description of the features of the proposed strategy will then be published in an open access peer-reviewed journal. We will also utilize graphics to disseminate core highlights of the strategy with our study populations and providers. We will plan a new study to undergo a real-world exploration of the strategy with our study populations.

### Data analysis

We will use van Manen’s thematic analysis of qualitative data [70]. Transcripts will be read in their entirety, then line by line, and then in search of evocative statements. In following an interpretive approach [70, 71] in the development of themes, we will focus on experiences shared by participants that suggest core threads of their lived experiences. We will use an iterative process of theme development. As data analysis progresses, preliminary themes are further developed or discarded until a thorough description of the theme is achieved. We will do manual data analysis and generate the themes through writing and rewriting. Each theme will portray elements of participants’ experiences of living with advanced cancer, access to palliative care, and inequities in access. We will also examine how the intersections of race, gender, socioeconomic status, and other social dimensions shape their experience.

Gender as a sociocultural factor will be a core element of data analysis. We will pay close attention to gender disparities as evidence points to important gender disparities within racialized communities and also between racialized and non-racialized populations [72]. Additional gender considerations will include gender roles and gender identity. Gender roles such as family caregiving, child care, household chores, and being the main provider in the household, among others, may prevent individuals from timely accessing health care and result in negative care outcomes. Precarious employment, lack of health and pension benefits, and financial needs may create delays in access to health care. While our study does not have an explicit focus on gender diverse communities, members of these communities who self-identify as Blacks or Latin Americans may be interested in participating in our study. We will thoroughly examine the gender experiences of our study participants and explore inequities associated with these. An understanding of how gender affects the lived experiences of our study population will generate rich insight into their experiences of access to cancer and palliative care and will help us further clarify how the intersections of gender with other social dimensions contribute to suboptimal health experiences.

### Ethical considerations

This research study was approved by the Health Research Ethics Board of Alberta Cancer Committee on December 22, 2022 (Ethics ID HREBA.CC-22–0351). We will strictly adhere to the Tri-Council Policy Statement on the conduct of research with human beings [73]. Study activities impose no foreseeable risks other than those participants could experience in their daily life and regular health care encounters. While there will be no direct benefits to study participants, risks associated with participation are minimal. Participation in the study will be voluntary. Deciding not to take part or deciding to leave the study early will not result in any penalty or affect current or future care or employment. Participants will also have the freedom to leave the study at any time without giving reason. All study data will be de-identified and anonymized prior to data analysis. We will keep research data for a minimum of 5 years or until the end of scholarly activity related to the study. All data will be secured stored in a password protected research directory at the first author’s home institution.

People with advanced cancer are highly vulnerable due to the frailty of their health condition. We will invite them to talk about their cancer journeys and access to cancer care services. Patient participants may feel fatigue during the interviews or may become emotional while talking about their experiences. Family members may also experience fatigue or become emotionally distressed when talking about their experiences of care. Community members may share personal experiences of knowing someone living with cancer, or who died of cancer. All participants may become tired or emotional when sharing personal experiences as members of racialized communities in Canada.

Our team brings solid skills in palliative care. We will take steps to minimize any undue distress. Questions will be worded in a respectful way and will be minimally intrusive. Should any conversation become emotionally taxing, we will stop the interview, stay with the study participant until settled, and offer to contact their health care provider if needed. Interviews will be limited to 45 to 60 min to minimize tiredness and could be shortened if needed. Patient burden will be a key consideration and we will approach individuals who are clinically stable.

All members of the study team including our patient partners will receive training in qualitative research. Our interviewers will bring lived or professional experiences working with members of our study populations and will have knowledge of equity, diversity, and inclusivity considerations in the conduct of research with racialized populations. To increase cultural safety, we will invite patient partners to co-interview study participants.

## Discussion

Health inequities are a pressing global concern that demand the collective engagement of world leaders and global citizens. This study is innovative in that it is the first of its kind to co-create together with participants ways of improving access to palliative care for patients with advanced cancer from Black and Latin American communities in Canada. Moreover, we employ community engagement and intersectionality frameworks, and will actively engage members of the Latin American and African communities through a patient engagement process. The study is also being undertaken by a robust team of researchers, scholars, patient partners, and health care providers with combined expertise in health equity, oncology and palliative care, Latin American and African scholarship, intersectionality, and qualitative research, and an understanding of the sociocultural identities of Latin American and African peoples.

Study outcomes include an increased knowledge of access to palliative care experiences of advanced cancer patients from racialized communities of African and Latin American descent in Canada and elements to increase access to palliative care and reduce inequities in these communities. This study will inform ways of improving access to palliative care for racialized communities in other parts of Canada and globally.

In following the principles of health equity and intersectionality, in this study we will employ a perspective that takes into account the concurrent impacts of several social categories including gender, race and racism, socioeconomic status, belonging to a racialized community, and language barriers, among others. Understanding the impacts of multiple intersections of social categories will help uncover the underlying mechanisms that contribute to inequities in access to palliative care for members of African and Latin American descent in Canada. Inequities in cancer and palliative care result and can be exacerbated by the compounding impact of interacting social dimensions. This understanding is critical to being able to improve equity.

The engagement of the PAC will be vital to facilitate a continuous knowledge exchange process between patient partners, researchers, oncology and palliative care providers, and implementers. Patient partners have collaborated with the development of interview guides, recruitment approach and materials, may engage as co-interviewers of study participants, and will engage in data analysis and interpretation to increase understanding and relevance of interpretation of participants’ experiences. This will contribute to the production of culturally relevant research findings and evidence, and will facilitate the dissemination of knowledge to the communities.

The main study output is the Access to Palliative Care Strategy for advanced cancer patients of African and Latin American descent. Additional study outputs include open-access publications, presentations with diverse stakeholders, and graphics to highlight key study findings. Patient engagement is at the core of our knowledge translation approach.

### Closing remarks

This protocol is subject to change as we follow the guidance of patient partners, community members, and the study team in the undertaking of this research. The study will generate knowledge to improve patients’ experiences of access to palliative care with a study population known to experience inequities in access to health care. We hope that study findings will inform future palliative care practice with these populations and contribute to reduce inequities in cancer care.

### Supplementary Information

**Additional file 1.**

**Additional file 2.**

## Data Availability

Not applicable.

## Abbreviations

ACSG: African Cancer Support Group
CCI: Cross Cancer Institute
HREBA: Health Research Ethics Board of Alberta
PAC: Patient Advisory Council
