# Supplementary material for: Increasing access to palliative care for patients with advanced cancer of African and Latin American descent: a patient-oriented community-based study protocol
Source: BMC Palliat Care. 2023 Dec 20;22:204. doi: 10.1186/s12904-023-01323-0 (PMC10731745; doi:10.1186/s12904-023-01323-0)
Supplement: Supplementary file 1 — Additional file 1. [file 12904_2023_1323_MOESM1_ESM.docx]

**Interview Questions for Patients**

**What can you tell us about your experiences of living with cancer?**

1. Tell us a little bit about yourself and your health experiences.
   - Probes:
   - Would you like to introduce yourself?
   - What is the most important thing about your cancer experience you would like to share?
2. What can you tell us about your experience of living with cancer?
   - Probes:
   - What was it like when you first learned that you had cancer?
   - What can you tell us about your journey since the time you began feeling unwell?
3. What is it like for you to access health care when living with cancer?
4. Can you describe your experiences of accessing palliative care?
   - Probes: For example, treatments, doctor’s appointments, pain management, emotional, social or financial supports?
5. Are there personal characteristics that, in your view, have affected your access to care?

- Probes: Race, gender, socioeconomic status, language, job status, place of residence, immigration status or other characteristics?

1. In your view, do you see any or a combination of these personal characteristics or living conditions affecting your access to care?
2. Is there anything that you would suggest to improve access to care for cancer patients?
3. Is there anything else that you would like to share that we have not discussed?

**What can you tell us about your diagnosis?**

- What is your cancer diagnosis?
- When were you diagnosed with cancer?
- When did you receive your cancer diagnosis?
- Where were you diagnosed with cancer?
- How did you receive your cancer diagnosis? (probe: who gave you the diagnosis)
- What cancer treatments have you received?
- Are you currently receiving palliative care?

Interview end time: __________________________

**Interview Questions for Family Members**

INTERVIEW START TIME: __________________

**What can you tell us about your experience as a family member of someone living with cancer?**

1. Tell us a little bit about yourself
   - Probes: What is your relationship to family member living with cancer?
2. What can you tell us about your experience as a family member of someone living with cancer?
   - Probes: What was it like when you first learned that your family member had cancer?
   - What can you tell us about your journey since the time your family member began feeling unwell?
3. In your view, what is it like for your family member to access health care when living with cancer?
4. How has the experience accessing care been like for you as a family member?

- Probes: Have you had any involvement making care decisions with or for your family member? If yes, what has that experience been like?

1. Can you describe their experiences of accessing palliative care? Or your experience accessing palliative care for a family member?
   - Probes: For examples, treatments, doctor’s appointments, pain management, emotional, social, or financial supports?
2. Are there personal characteristics that, in your view, have affected their access to care? For example, race, gender, socioeconomic status, language, job status, place of residence, immigration status or other characteristics?
3. In your view, do you see any or a combination of these characteristics affecting their access to care?
4. Is there anything that you would suggest to improve access to care for cancer patients?
5. Is there anything else that you would like to share that we have not discussed?

INTERVIEW END TIME: __________________________

**Interview Questions for Community Members**

INTERVIEW START TIME: __________________

**What can you tell us about your perspectives on access to palliative care for people of African or Latin American descent living with cancer?**

1. Tell us a little bit about yourself.
2. What can you tell us about your perspectives on access to palliative care for people of African or Latin American descent living with cancer?
   - Probes: For example, treatments, doctor’s appointments, pain management, emotional, social or financial supports?
3. What does palliative care mean in your community? (e.g. African/Latin American Community) And does the meaning influence access to care?
4. In your view, what is it like for members of your community (African or Latin American) to access health care when living with cancer?
5. Have you had any experiences with family, friends, or other community members accessing palliative care when living with cancer? How would you describe their experiences?
6. Are there personal characteristics that, in your view, can affect African or Latin American people’s access to care? For example, race, gender, socioeconomic status, language, job status, place of residence, immigration status, or other characteristics?
7. Have you seen any issues with housing, transportation, or other living conditions that could affect their access to care?
8. In your view, do you see any or a combination of these personal characteristics or living conditions affecting their access to care?
9. Is there anything that you would suggest to improve access to care for cancer patients?
10. Is there anything else that you would like to share that we have not discussed?

Interview end time: __________________________
