# Supplementary material for: Increasing access to palliative care for patients with advanced cancer of African and Latin American descent: a patient-oriented community-based study protocol
Source: BMC Palliat Care. 2023 Dec 20;22:204. doi: 10.1186/s12904-023-01323-0 (PMC10731745; doi:10.1186/s12904-023-01323-0)
Supplement: Supplementary file 2 — Additional file 2. [file 12904_2023_1323_MOESM2_ESM.docx]

**Preliminary Guide for Virtual Conversations with Health Care Providers**

**Questions for health care providers**

- What are your perspectives on the strategy? What changes would you make?
- In your view, is the strategy suitable to serve people of African or Latin American descent living with advanced cancer?
- Do you see the strategy having an impact on people’s access to palliative care? In what ways?
- Do you anticipate any risks associated with the implementation of the strategy?
- Is it convenient? How so?
- What would be minimum requirements to implement the strategy in your health care setting?
- What barriers do you anticipate for its successful implementation?

end time: __________________________
